# The diabesity health economic crisis—the size of the crisis in a European island state following a cross-sectional study

**DOI:** 10.1186/s13690-016-0164-6

**Published:** 2016-12-12

**Authors:** Sarah Cuschieri, Josanne Vassallo, Neville Calleja, Nikolai Pace, Janice Abela, Bader A. Ali, Fatemah Abdullah, Elizier Zahra, Julian Mamo

**Affiliations:** 1 Department of Anatomy, Faculty of Medicine and Surgery, Biomedical Building, University of Malta, Msida, MSD 2080 Malta; 2 Faculty of Medicine and Surgery, University of Malta, Msida, Malta; 3 Department of Public Health, Faculty of Medicine and Surgery, University of Malta, Msida, Malta; 4 Faculty of Dental Surgery, University of Malta, Msida, Malta; 5 Mater Dei Hospital, Msida, Malta

**Keywords:** Economic burden of disease, Burden of illness, Diabetes mellitus, Obesity, Malta

## Abstract

**Background:**

Diabetes type 2 and obesity are well-established global epidemics and contributors to clinical, social and economic health burdens. The prevalence rates of these diseases are still on the rise among countries resulting in a corresponding public health burden. The Mediterranean island of Malta, known for it’s high diabetes and obesity rates, provides a good fundamental basis to portray the economical health burden of these diseases.

**Method:**

A recent randomised stratified representative cross-sectional survey conducted in Malta tackling diabetes, obesity and other determinants, was used to work out the population prevalence of these diseases. The cost burden of diabetes and obesity, based on published data, was incorporated to the established population prevalence rates, in order to estimate the Maltese economical burden. Projections to the year 2050 by a bottom-up prevalence based design were performed.

**Results:**

One eight of the Maltese adults (25 to 64 years) suffered from diabetes out of which approximately 10,000 adults were unaware of the disease. Alarmingly, more than a third of the Maltese population suffer from obesity. The approximate health care costs (direct and indirect) for the diabetic adult population was of €29,159,217 (€21,994,676 - €38,919,121) annually, amounting to 3.64% (2.75–4.875%) of the total health expenditure in Malta. The obesity cost burden was of €23,732,781 (€21,514,972-€26,049,204) annually contributing for 2.97% (2.69–3.26%) of the total health expenditure. The projected prevalence and costs for 2050 exhibited an estimated cost burden increase of €33,751,487 (€25,458,606–€45,048,473) for the diabetes mellitus population and €46,532,294 (€42,183,889–€51,074,049) for the obese population. These projected cost burdens are expected to increase exponentially the total health care expenditure in Malta by 2050.

**Conclusion:**

Having an understanding of the prevalence and the economic cost burden of diabetes and obesity within a country, along with projections of the expected burden will enable policy and public health officials to clearly visualize this growing problem. It also helps in establishing effective preventive strategies and screening programs targeting these epidemics.

## Background

The global obesity and diabetes mellitus type 2 prevalence rates have increased exponentially over recent years [1, 2]. Globally an estimated 2 billion people are overweight, a third of which being obese [1]. The increasing prevalence of obesity is predicted to lead to other chronic related diseases [3]. The link between obesity and type 2 diabetes is well established through the development of obesity related insulin resistance. It follows that the prevalence of the two diseases is directly proportional [3]. However, only about 20–25% of the overweight and obese population is expected to develop diabetes [4]. Both diseases lead to heavy increases in clinical, social and economic health burdens as well as to increments in morbidity and mortality [5, 6]. They also impact on employment and productivity, both of which have significant direct impacts on a country’s productivity and economical growth [7].

Malta is a European island nation in the middle of the Mediterranean Sea with a total population of 425,384 (median age 40.9 years). Its history is littered with diverse dominating powers and shifts in its culture throughout the centuries. Up to 150 years ago, the population followed a Mediterranean lifestyle and diet, although this has now shifted to a more Westernized lifestyle and diet [8]. The World Health Organization (WHO) conducted the last Maltese national prevalence study in Diabetes in 1981 [9]. Subsequently, a health examination pilot study was conducted on the Island in 2010. However, the latter study had a small sample size (*n* = 221) and offered only a glimpse of the actual diabetes and obesity burden [10]. This has led to Maltese policies and economic burden extrapolations being based on outdated sources, despite strong emigrational, cultural and lifestyle changes over recent decades [8]. Between 2014 and 2016, the University of Malta “SAHHTEK” (*Your Health*) cross-sectional study was conducted. This provided valid, updated prevalence rates, through which the diabetes mellitus type 2 and obesity health burden in Malta could be established. The aim of this study was to establish the current (for 2016) prevalence and economic burden of diabetes type 2 and obesity in Malta. The objectives were: 1) to establish the body mass distribution within the diabetic sub-population by age and gender; 2) to estimate the projected total population prevalence of type 2 diabetes and obesity in Malta for 2050; 3) estimate the economic burden of diabetes and obesity for the year 2050 and; 4) provide evidence based data for policy makers to target and implement prevention strategies concerning these non-communicable diseases. This study will serve as a fundamental basis for the burden estimation in neighbouring Mediterranean countries.

## Method

A cross-sectional health examination survey was conducted between November 2014 and January 2016. The sample population was obtained from the national registry after performing randomization and stratification of the data by age (18 to 70 years) and gender [11]. The data was further stratified according to residence locality, to represent an approximate 1% population from each Maltese town. The total randomized and stratified population (*n =* 4,000) was invited to participate in a health examination survey set up in every Maltese town through an invitation letter sent out via post. The health examination survey consisted of a validated questionnaire and health examination including weight and height as well as blood letting for fasting blood glucose and lipid profile.

The measured weight and height was used to calculate the body mass index (BMI) by dividing the weight (in kilograms - kg) over the height squared (meters squared - m^2^). Those participants with a BMI <24.99Kg/m^2^ were considered as normal BMI; 25–29.99Kg/m^2^ as overweight and >30Kg/m^2^ as obese [12]. Participants were considered as suffering from ‘*previously known diabetes mellitus*’ if they gave a medical history of diabetes or were on oral hypoglycemic agents, irrelevant of the measured fasting blood glucose. Meanwhile, all those obtaining measured fasting blood glucose above 7 mmol/L were considered as newly diagnosed diabetics [13].

The diabetes population (total) was defined as the sum of the previously known diabetes mellitus participants to the newly diagnosed participants. Prevalence rates of diabetes (by gender and 10-year age groups) were calculated by dividing the total diabetes population by the total participants who attended the study. The prevalence rate was applied to the latest officially recorded total Maltese population demographic statistics to obtain the overall population with diabetes [12]. Similar calculations were used to estimate the prevalence rates of those with normal, overweight or obese status in the Maltese population [14, 15]. Sensitive analyses based on the confidence intervals of the prevalence rates were preformed. The study only considered type 2 diabetes mellitus individuals. The Research Ethics Committee of the Faculty of Medicine and Surgery at the University of Malta together with the Information and Data protection commissioner gave their consent for this study.

The estimated cost burden for diabetes in Malta was calculated by applying the established prevalence rate of *newly diagnosed diabetics* and that for the *previously known diabetics* (individually) to the cost burden found in the literature for each diabetes subgroup. The cost burden for the newly diagnosed diabetics population was based on estimates performed by Zhang Y et al. (2009) in the USA after adjusting for the gross domestic poduct (GDP) per capita and by adjusting for deflation between the USA and Malta [16]. The cost burden for the *previously known diabetes population* was obtained from published International Diabetes Federation (IDF) data on Malta [17]. The cost attributed for the obese population was obtained from a study conducted in Malta by the Department of Health Information and Research in 2008, based on the 2008 European Health Information Survey, after 2% compound interest per annum was performed [18].

Projections to the year 2050 were performed by a bottom-up prevalence-based design for diabetes and for obese rates (1981 to 2014–2015 surveys). For purposes of comparative and statistical analysis, only the subgroup of adults between 25 and 64 years of age were utilized from SAHHTEK. This was required to keep a valid comparison with the 1981 study. By hypothesizing that no other factors distorted linear projections for both disease prevalence rates, an estimation of the affected population and their economic burden was established based on the EUROSTAT 2050 population projections for Malta [19]. Statistical analysis including chi squared was calculated by means of IBM SPSS version 21. Demographic and lifestyle data was gathered by means of a combination of validated tools used during the “SAHHTEK” survey [20–22].

## Results

The sample population that attended the study (response rate of 49% *p* = <0.05) was weighted according to age, gender and locality. This enabled the data to be statistically representative of the whole population of Malta as well as to take in consideration the non-responders. After statistically weighting the sample, the final population dataset was of 3,947 (1998 males, 1949 females). Out of which, 10.39% (95% CI: 9.47–11.38) suffered from diabetes, among which 6.31% (95% CI: 5.59–7.11) were previously known diabetics and 4.08% (CI: 3.50–4.74) were newly diagnosed. 69.77% (95% CI: 68.32–71.18) of the total population was found to be either overweight or obese (Table 1) with a heavy male predominance. The diabetic population was predominantly either overweight or obese (92.20% CI 95%: 89.16–94.45) as seen in Table 1.

**Table 1:** BMI prevalence rates by population (diabetic and non-diabetic) population

BMI category			
Total Population	Normal % (95% CI)	Overweight % (95% CI)	Obese % (95% CI)
Total (*n* = 3947)	30.26% (28.84–31.70)	35.66% (34.17–37.15)	34.11% (32.64–35.60)
Male (*n* = 1998)	23.72% (21.91–25.64)	39.39% (37.27–41.55)	36.89% (34.80–39.03)
Female (*n* = 1949)	36.94% (34.83–39.11)	31.81% (29.79–33.91)	31.25% (29.23–33.34)
*p*-value*	0.0001	0.0001	0.0001
Diabetic Population	Normal % (95% CI)	Overweight % (95% CI)	Obese % (95% CI)
Total (*n* = 410)	7.8% (5.55–10.84)	36.83% (32.30–41.60)	55.37% (50.53–60.11)
Male (*n* = 274)	8.39% (5.61–12.33)	39.78% (34.16–45.68)	51.82% (45.92–57.57)
Female (*n* = 136)	6.62% (3.36–12.26)	30.88% (23.71–39.10)	62.50% (54.12–70.19)
*p*-value*	0.73	0.84	0.327
Non-Diabetic Population	Normal % (95% CI)	Overweight % (95% CI)	Obese % (95% CI)
Total (3537)	32.85% (31.31–34.42)	35.51% (33.76–36.90)	31.64% (30.12–33.19)
Male (1724)	26.16% (24.14–28.29)	39.33% (37.05–41.65)	34.51% (32.31–36.79)
Female (*n* = 1813)	39.22% (36.99–41.48)	31.88% (29.78–34.06)	28.9% (26.86–31.03)
*p*-value*	0.0001	0.0001	0.0001

*Chi squared between each BMI category against gender

It was noted that younger (<55 years) diabetics were predominately (54% CI 95%: 44.30–63.12%) new (unknown) diabetics, while for those aged 55 and over, only 34% (CI 95%: 29.21–39.80) were new diabetics with the vast majority (66% CI 95%: 60.20–70.79) being known diabetics. This finding is in keeping with published data evaluating the degree of diabetes awareness between the 1981 and 2010 examination surveys conducted in Malta [23].

Further subdivision of the diabetic population by age and body weight revealed younger diabetics (<55 years) to be predominately obese (50% CI 95%: 40.56–59.44) or overweight (46% CI 95%: 36.88–55.70), with only 4% (CI 95%: 2–8.9) having normal weight. Conversely, a higher proportion of older diabetics (≥55 years) were found to belong to the normal weight category (9% CI 95%: 6.37–12.50) with 57% (CI 95%: 51.50–61.61) of these found obese and 34% (CI 95%: 28.59–39.13) found to be overweight (*p* = 0.001).

On incorporating the established prevalence rates and their corresponding confidence intervals from this study to the Maltese population, an estimate of the present total population burden of diabetes mellitus and obesity by 10-year age groups and gender was established [14]. Table 2 shows the estimated total Maltese diabetes population by gender and age.

**Table 2:** Estimation of the diabetes mellitus population burden by applying the prevalence rates to Maltese population data by age and gender

Maltese Population Diabetes Prevalence				
Age	Total Maltese Population by age^a^	Total (CI)	Previously Known (CI)	Unknown Diabetics (CI)
25–34	62,180	404 (143–964)		404 (143–964)
35–44	56,575	2,489 (1,765–3,491)	933 (515–1,641)	1,556 (996–2,399)
45–54	55,113	5,054 (4,007–6,332)	2,712 (1,962–3,726)	2,337 (1,642–3,296)
55–64	59,268	11,611 (10,052–13,347)	6,638 (5,435–8,060)	4,976 (3,935–6,253)
Total	233,136	19,558 (15,967–24,134)	10,283 (7,912–13,427)	9,273 (6,716–12,911)
Age	Male Maltese Population by age^a^	Maltese Male Population Diabetes Prevalence		
		Total (CI)	Previously Known (CI)	Unknown Diabetics (CI)
25–34	32,359			
35–44	29,143	1,781 (1,195–2,611)	667 (332–1,271)	1,113 (664–1,821)
45–54	27,728	4,204 (3,252–5,368)	2,426 (1,708–3,397)	1,777 (1,173–2,651)
55–64	29,585	6,935 (5,784–8,236)	3,763 (2,896–4,840)	3,172 (2,379–4,189)
Total	118,815	12,920 (10,231–16,216)	6,856 (4,937–9,507)	6,062 (4,216–8,661)
Age	Female Maltese Population by age^a^	Maltese Female Population Diabetes Prevalence		
		Total (CI)	Previously Known (CI)	Unknown Diabetics (CI)
25–34	29,821	397 (143–945)		397 (143–945)
35–44	27,432	658 (313–1,300)	247 (49–749)	411 (148–977)
45–54	27,385	1,060 (633–1,733)	424 (173–934)	630 (318–1,210)
55–64	29,683	4,625 (3,648–5,806)	2,853 (2087–3,856)	1,772 (1,178–2,624)
Total	114,321	6,740 (4,736–9,785)	3,524 (2,309–5,539)	3,210 (1,787–5,756)

CI − 95% Confidence intervals

^a^2013 Malta Demographic Report

In the Maltese adult population between 25 and 64 years of age, approximately 20,000 adults suffer from diabetes mellitus type 2. Out of which, approximately 10,000 adults suffer from diabetes and are yet unaware of it, with a male predominance (approx. 6,000). On comparing the Maltese diabetes population to the IDF Atlas (7^th^ Edition) European diabetes population, the Maltese diabetic population contributes to an approximate 0.03% of diabetes within Europe [2]. This prevalence percentage is an estimate comparison since the adult population age groups for the IDF (20 to 79 years) was different when compared to SAHHTEK study (25 to 64 years).

On the other hand, approximately 82,000 of the 233,136 adults (35% of the adult population) in this age category (25–64 years) in Malta are obese, with a persisting male predominance (approx. 46,000) (Table 3). This ranks the Maltese population as the most obese country in the Mediterranean (Italy – 10.5%; Cyprus – 13.9%; Greece – 16.9%) and in Europe [24].

**Table 3:** Estimation of the obese population burden by applying the prevalence rates to Maltese population data by age and gender

Maltese Population		
Age	Total Maltese Population by age^a^	Obese BMI (CI)
25–34	62,180	15,968 (14,127–17,951)
35–44	56,575	18,523 (16,644–20,497)
45–54	55,113	22,315 (20,386–24,299)
55–64	59,268	25,260 (23,239–27,328)
Total	233,136	82,066 (74,397–90,076)
Age	Male Maltese Population by age^a^	Obese BMI (CI)
25–34	32,359	9,569 (8,187–11,076)
35–44	29,143	12,310 (10,914–13,750)
45–54	27,728	12,611 (11,172–14,078)
55–64	29,585	11,730 (10,349–13,171)
Total	155,996	46,220 (40,621–52,075)
Age	Female Maltese Population by age^a^	Obese BMI (CI)
25–34	29,821	6,423 (5,272–7,747)
35–44	27,432	5,914 (4,792–7,212)
45–54	27,385	9,881 (8,615–11,222)
55–64	29,683	13,568 (12,111–15,052)
Total	114,321	35,786 (30,791 - 41,234)

CI - 95% Confidence intervals

^a^2013 Malta Demographic Report

Estimating the cost burden (both direct and indirect) of an undiagnosed diabetic to be approximately €1,052 per person per year (6.67% of the annual mean salary income per person in Malta), the annual burden for the entire population (25 to 64 years) was estimated at €9,755,196 (€7,065,232–€13,582,372), which contributes to 1.22% (0.88–1.70%) of the total health expenditure for Malta [16, 25]. The total health expenditure for Malta (for 2016) is approximately €800,000,000 [25]. The direct medical costs include hospital inpatient costs, physician care, emergency care, outpatients care and prescriptions, while the indirect costs included absence from work, reduced work performance and productivity [16]. Conversely, a known diabetic person’s disease cost burden was approximately €1,887 per person annually (11.96% of the annual mean salary income per person in Malta), attributing to an annual health burden of €19,404,021 (€14,929,944–€25,336,749) for the entire population (25 to 64 years) [17]. This is consistent with 2.43% (1.87–3.17%) of the Maltese total health expenditure. Therefore the global cost burden of diabetes mellitus for the Maltese health system is approximate a total of €29,159,217 (€21,994,676–€38,919,121) annually. The diabetes cost burden contributes to 3.65% (2.79–4.87%) of the total health expenditure (state and private expenditure) for Malta [25]. On the other hand, the attributed cost burden (included inpatient stay, day patient stay, general practitioner and specialist consultations but not medication and surgical procedures) for obesity in Malta (2% compound interest per annum), was estimated to be €23,732,781 (€21,514,972–€26,049,204) for the year 2016 [18]. The obesity cost burden contributes to 2.97% (2.69–3.26%) of the total health expenditure for Malta [25]. Therefore the total ‘diabesity’ cost burden for Malta, in 2016, is an approximate total of €52,891,998 (€43,509,648–€64,968,325) which amounts to 6.61% (5.44–8.12%) of the Malta’s total health expenditure.

The projected EUROSTAT 2050 Maltese population (25 to 64 years) was incorporated within the projected prevalence rates for diabetes and obesity rates as seen in Table 4. On incorporating these projected prevalence rates within the projected cost burden (2% compound interest per annum from the 2016 costs) for diabetes and obesity, we were able to estimate the likely economic burden caused by these diseases for 2050 (Table 5). The total adult population (25 to 64 years) projected for 2050 appears to decrease from the current 2016 adult population. Meanwhile, the diabetes and obese population will increase by approximately 28 and 15% respectively by 2050. The economic burden is expected to increase in association with this disease burden. An exponentiation of 1.2 in the diabetes cost burden and exponentiation of 2 in obese cost burden from the current (2016) is expected to occur by 2050. This contributes to an estimated cost burden of €33,751,487 (€25,458,606–€45,048,473) for diabetes mellitus and €46,532,294 (€42,183,889–€51,074,049) for the obese. Therefore the estimated total cost burden for 2050 for diabetes mellitus type 2 and obesity would amount to one-eight of the current (2016) total health expenditure (€800,000,000).

**Table 4:** Projected prevalence rates of diabetes and obese population for 2050 in Malta

Age	2050 Projected Total Maltese Population by age^a^	Projected Diabetes & Obesity Prevalence for 2050	
		Total Diabetes (CI)	Obese (CI)
25–34	56,709	601 (255–913)	21,810 (19,791–23,829)
35–44	52,965	3,813 (2,961–4,666)	21,175 (19,650–22,685)
45–54	53,819	5,457 (4,698–6,211)	22,970 (21,361–24,579)
55–64	62,342	15,199 (14,014–16,153)	28,079 (26,882–29,295)
Total	225,835	25,071 (21,929–27,943)	94,034 (87,684–100,388)

CI - 95% Confidence interval

^a^Demographic Data 2013

**Table 5:** Comparison of the total cost burden for diabetes and obese population for 2016 and those projected for 2050

Maltese Population (25 to 64 years)		
	Total Diabetes	Obese
2016 Costs	€29,159,217	€23,732,781
	(€21,994,676–€38,919,121)	(€21,514,972–€26,049,204)
2050 Projected Costs with 2% Compound interest per annum	€33,751,487	€46,532,294
	(€25,458,606–€45,048,473)	(€42,183,889–€51,074,049)

## Discussion

Diabetes mellitus type 2 and obesity prevalence rates are on the rise with the majority of diabetic patients being obese [26]. This is observed in the current study where the bulk of the Maltese population was either overweight or obese. Apart from this, a tenth of the total adult population (18 to 70 years) suffered from diabetes mellitus type 2. Another worrying feature is that the majority of the Maltese diabetic population was obese. The projected Maltese estimates for 2050, also exhibit an exponential increase in the obese population, along with a drastic increase in diabetes mellitus. Although diabetes might not develop in all those who are obese, the accumulation of adipose tissue initiates a cascade of metabolic events. These in turn have the ability to initiate or exacerbate insulin resistance in those more predisposed. All this can subsequently lead to the development of various metabolic diseases including hypertension, dyslipideamia and cardiovascular disease [27].

Tackling the obesity epidemic in its own right is of utmost importance in order to prevent further obesity-related illnesses that are contributing not only to a decrease in the quality of life and mortality of many, but also attributing to an estimated 8 to 10% of the total health expenditure in many European countries [28]. In Malta, the cost burden attributed to overweight-obesity was estimated to be 5.7% of total health expenditure in 2008 [18]. Considering that only the obesity burden was evaluated in the current study, one cannot directly compare to the 2008 (Malta) health expenditure. However, no drastic exponential increase appears to have occurred. On comparing the current obesity impact on the total health expenditure to other European countries, Malta relates well to the obesity related expenditure reported [28].

The International Diabetes Federation estimated the 2009 cost burden for diabetes mellitus to contribute to 11% of the total health expenditure in Malta [29]. Our results showed that diabetes (direct and indirect) cost burden on the total health expenditure has decreased by approximately three-fold from the IDF estimate for Malta. According to the IDF, diabetes mellitus type 2 accounts for 5 to 20% of the majority of the countries’ total health care expenditure, which is in keeping to the study’s results [2]. In Malta, diabetes appears to have the same burden effect on the total health care expenditure as other countries.

The diabetes and obesity epidemics present as a great burden of care and an economic challenge to the Maltese population from the relatively young age of 35 years onwards [30]. In fact, extrapolations from the current (2016) obese and diabetes burden to 2050, exhibit a major health care concern for Malta, especially with the expected high diabetes prevalence rise and related economical costs. It is questionable whether the overall health budget will also swell proportionately, given the current economic situation. This is more so considering that by 2050, diabetes and obesity health burdens are expected to amount to one-eight of the current (2016) total health care expenditure. The planned Malta EU Presidency for 2017 (January to June) reflects the obesity burden and is planned to tackle Childhood obesity. Considering that a substantial proportion of the young population (<55 years) was found to have unknown diabetics and an increased body weight (obese > overweight), this brings forward the need for immediate action. The higher the obesity and diabetes prevalence rates, the higher the expected hospitalization rates and other related costs [5]. It is well established that the risk of co-morbidity increases with the increase in body mass index (BMI) [31]. Per capita costs reach their maximum among the obese population [32]. This phenomenon has been recorded in various European countries [33–35]. It is essential that health services in Malta and elsewhere in Europe embark on effective preventive strategies and opportunistic screening to target these conditions from an early age, before the disease has been ingrained for too long [23]. Although this could impose an even larger health budget request on the country, such early action could help decrease the health burden contributed to diabetes, obesity and their associated complications. In fact, disease prevention should always be the primary target of any health management strategy.

### Study limitations

The response rate obtained was 49%. This can present as a potential non-response bias but the data was statistically weighted according to age, gender and locality in order to overcome this potential bias. The study does not cover the whole population but only a subset of the adult population. Since the prevalence of diabetes is substantial within the elderly population, costs may be affected by other underlying comorbidities. General demographic data was based on the latest published reports from 2013. The health cost for undiagnosed diabetes was based on a U.S. study, as no data was available for the Maltese population, although the GPD per capita for both countries was taken in consideration. The cost of illness data was obtained from secondary sources and this may affect the validity of the cost of illness in the Maltese health care setting. The previously known diabetes cost was obtained from the 2015 IDF estimated costs for Malta but no detailed description was available to the health care costs constitution. All costs from the secondary sources lacked confidence intervals; therefore sensitivity analysis could not be performed on the unit costs. Comparisons with other studies were difficult due to the fact that the age groups and the total population samples were different from the current study and not comparable. Projections for the year 2050 were based on current conditions with an assumption that all demographic and risk factors would continue at their current rates. The economical burden excluded society burden including intangibles from pain and decreased quality of life, as well as the impact on caregivers.

## Conclusion

The study explored the present disease burden and economic costs for diabetes and obesity in Malta as well as those projected for 2050. A substantial prevalence and economical burden increase was estimated for 2050. The current Maltese health care expenditure for both diseases was similar to other European countries estimates. The data obtained should encourage policy makers to further explore the situation and bring forward preventative strategies considering the present ‘diabesity’ burden and the expected exponential increase. Implementing early screening tests for diabetes and obesity might be one of the best strategies to counteract the epidemic and reduce the overall costs.

## Abbreviations

BMI: Body mass index
GDP: Gross domestic product
IDF: International diabetes federation
WHO: World Health Organization
